# Hyperleukocytosis and Pseudohyperkalemia in a Patient With Severe SARS-COVID 19 Unmasks Chronic Lymphocytic Leukemia

**DOI:** 10.7759/cureus.20826

**Published:** 2021-12-30

**Authors:** Sasmith R Menakuru, Adelina Priscu, Amir Beirat, Vijaypal S Dhillon, Ahmed Salih, Joseph Emran

**Affiliations:** 1 Internal Medicine, Indiana University Health Ball Memorial Hospital, Muncie, USA

**Keywords:** hyperlymphocytosis, pseudohyperkalemia, cll complications, chronic lymphocytic leukemia (cll), covid-19

## Abstract

Coronavirus disease 2019 (COVID-19) has caused significant morbidity and mortality in a vast majority of the patient population, especially those with malignancies. Chronic lymphocytic leukemia (CLL) is the most common leukemia in adults and is often an indolent disease. High white blood cell counts greater than 120 k/cumm in chronic lymphocytic leukemia may be implicated in cases of COVID-19. Hyperleukocytosis leads to falsely elevated potassium levels due to cell fragility. Pseudohyperkalemia occurs when elevated potassium is present due to potassium movement out of cells during or after a blood sample is drawn. Pseudohyperkalemia may be suspected when elevated potassium is present in asymptomatic patients with no corresponding electrocardiogram findings. The authors present a case of hyperleukocytosis and pseudohyperkalemia in a patient whose COVID-19 infection unmasked CLL.

## Introduction

Coronavirus disease 2019 (COVID-19) caused by the novel coronavirus severe respiratory syndrome coronavirus 2 (SARS-CoV-2) has given rise to a plethora of symptoms in multiple organ systems, with hyperleukocytosis being a rare complication in patients with chronic lymphocytic leukemia (CLL) [1]. Chronic lymphocytic leukemia is usually an indolent disease where most patients will not require immediate treatment and can be monitored. However, in patients with concurrent COVID-19 and CLL, substantially elevated lymphocytes can occur in contrast to typical lymphopenia associated with COVID-19 [2]. Pseudohyperkalemia or artifactual hyperkalemia is seen secondary to red cell hemolysis occurring during or after a blood draw has occurred. High white blood cell counts greater than 120,000 can cause falsely elevated potassium concentrations due to the fragility of the cells due to mechanical stress. The clinical significance is that it can lead to unwarranted administration of potassium-lowering medication or emergency dialysis, which can worsen the outcomes [3]. The authors report a case of COVID-19 inducing hyperleukocytosis with a lymphocyte count of 200 k/cumm on admission in a patient with newly unmasked CLL and coexisting pseudohyperkalemia due to increased cell fragility.

## Case presentation

**Figure 1:**
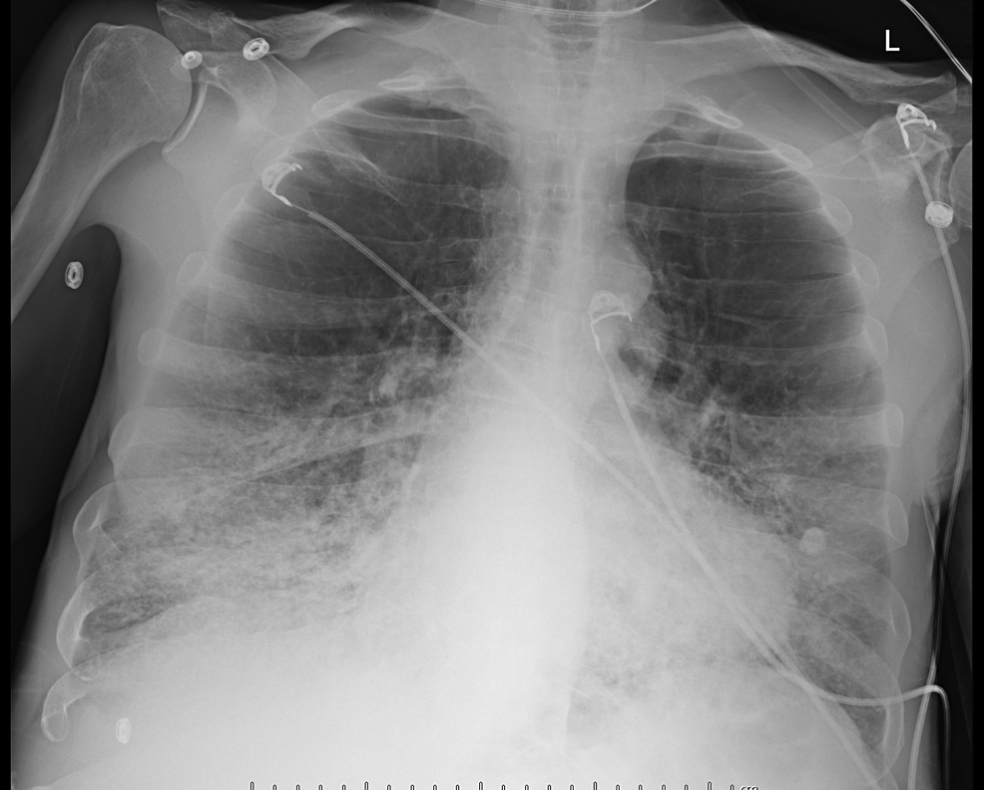
Radiography showing bilateral airspace disease typical of COVID-19

A 64-year-old male presented to the hospital with shortness of breath, a past medical history of hypertension, and chronic obstructive pulmonary disease. He was asymptomatic prior to presentation and did not have evidence of lymphadenopathy on examination. During his last primary care visit two weeks earlier, his WBC count was 13.9 k/cumm (3.6-10.6) and his absolute lymphocyte count was 5.8 k/cumm (1.0-3.2). He was asymptomatic prior to presentation and had no evidence of lymphadenopathy on examination. However, on admission he was diagnosed as positive for COVID-19 by polymerase chain reaction and had an oxygen saturation of 85% that required 11 liters of oxygen through a nasal cannula. A chest X-ray was taken which showed diffuse interstitial opacities with the appearance favoring COVID-19 [Figure 1].

**Figure 2:**
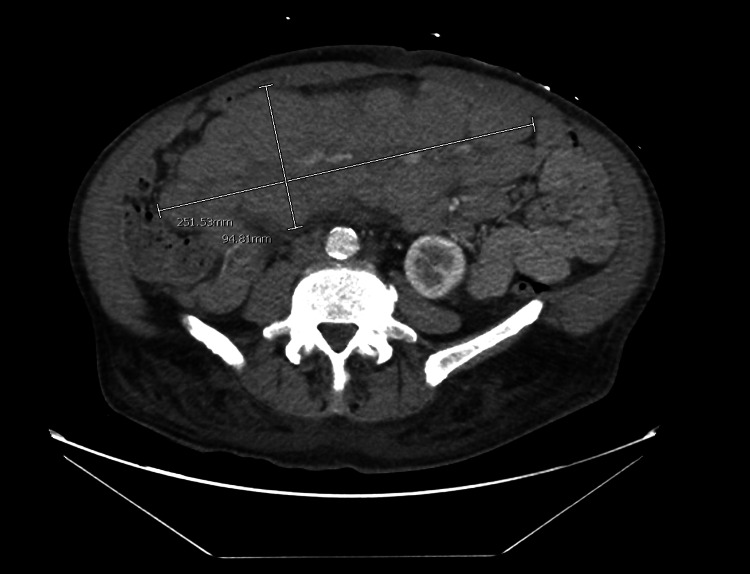
A CT scan of the chest reveals massive confluent lymphadenopathy in the mesentery

A CT chest was done to rule out pulmonary embolism which incidentally showed extensive adenopathy with enlarged lymph nodes within the bilateral supraclavicular fossa, bilateral axilla, mediastinum, and bilateral hilum, with the largest mass anterior to the abdominal aorta. The largest area of lymphadenopathy was located in the mesentery measuring 25.1 x 9.5 x 20.2 cm [Figure 2].

**Figure 3:**
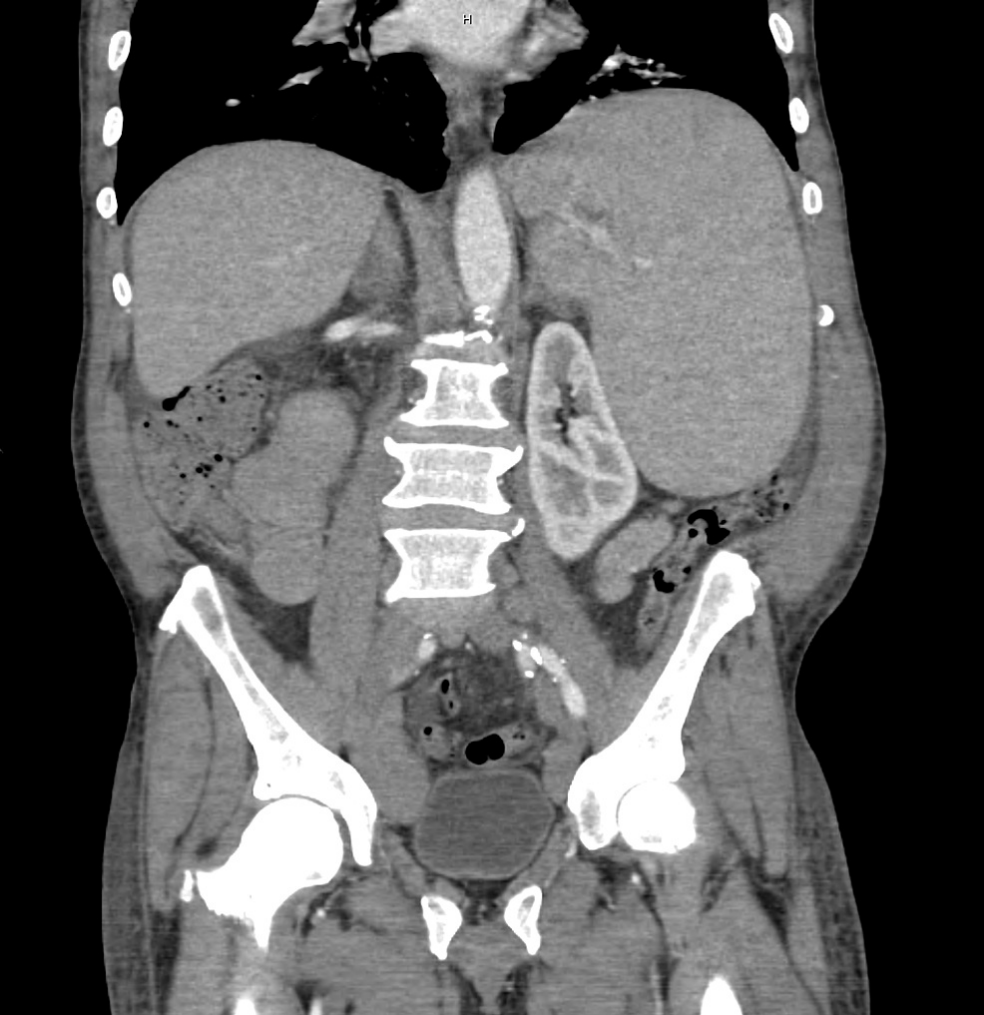
The CT scan of abdomen and pelvis shows bulky lymphadenopathy throughout with massive splenomegaly

A CT scan of the abdomen and pelvis showed bulky lymphadenopathy throughout with massive splenomegaly up to 22 cm and hepatomegaly [Figure 3].

**Figure 4:**
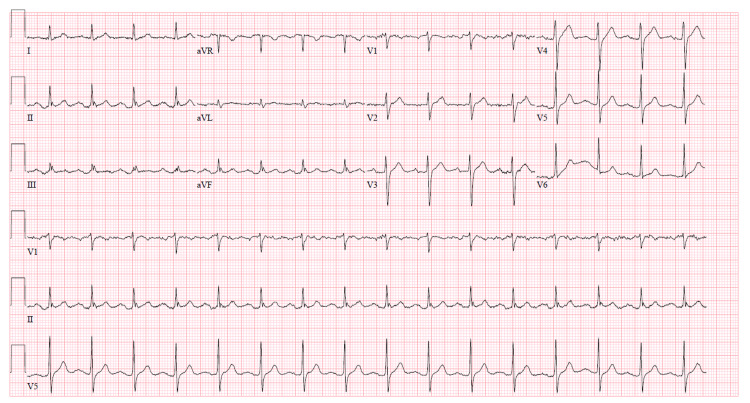
The ECG showing normal sinus rhythm

The electrocardiogram (ECG) showed normal sinus rhythm and did not show any pertinent findings [Figure 4]. 

**Figure 5:**
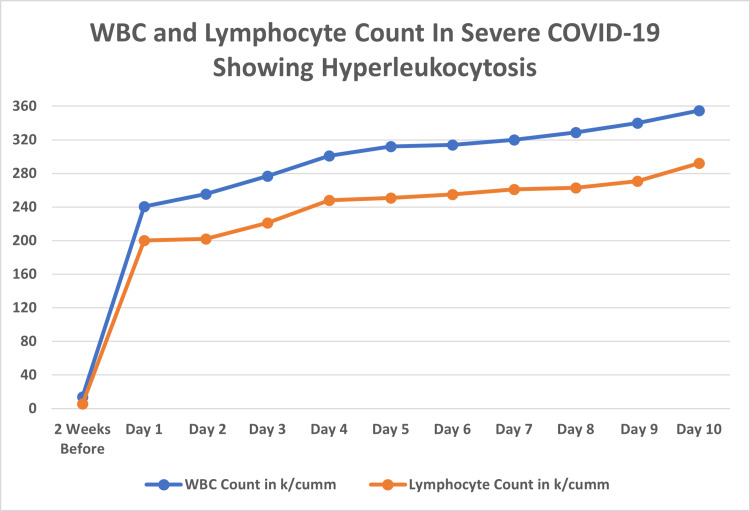
The WBC and leukocyte count two weeks before admission compared to day one of COVID-19 diagnosis. There was also a steady rise in both WBC and leukocyte counts each day.

The patient’s initial vital signs demonstrated a temperature of 38.1 C, a heart rate of 100 beats per minute, blood pressure of 150/78 mmHg, and a respiratory rate of 24 breaths per minute. Examination revealed an elderly appearing male in mild distress with an increased work of breathing. Pertinent laboratory investigations revealed a WBC count of 240.4 k/cumm (3.6-10.6), absolute lymphocyte count of 200.0 k/cumm (1.0-3.2), 3% blasts (< 0), potassium greater than 10.0 mmol/L (3.5-5.5), uric acid of 7.6 mg/dL (3.5-8.0), brain natriuretic peptide of 542 pg/mL (0-100), D-Dimer of 359 ng/mL (< 292), and high sensitivity troponin of 17 ng/L (0-20). The patient’s hyperleukocytosis and absolute lymphocyte counts steadily rose during the patient’s admission [Figure 5]. The patient was diagnosed with CLL as flow cytometric analysis at the time showed a monoclonal B-cell population with an expression of CD19, CD20, CD22, CD23, CD5, and kappa light chain consistent with CLL.

Given the elevation of potassium and lymphocytes with lack of ECG changes, suspicion was high for pseudohyperkalemia and therefore potassium was rechecked without the use of pneumatic tubes and centrifugation. The tube utilized for the repeat draw was not heparinized and adequate time was given for the separation of serum. The repeat potassium revealed a value of 5.3 which is a stark difference compared to the previous value confirming our suspicion for pseudohyperkalemia. 

The decision was made to treat COVID-19 first and then to focus on CLL. Remdesivir, a broad-spectrum antiviral, was administered at 200 mg on the first day as a loading dose and then 100 mg on subsequent days. Barticinib, a Janus kinase inhibitor, was also started at 4 mg daily, as it has been shown to be effective in severe cases of COVID-19. Dexamethasone 6 mg IV was also given daily. The patient’s respiratory function worsened and required bilevel positive airway pressure (BiPAP) by day three. The patient’s prognosis was made aware to the family as the possibility of intubation was imminent. However, the patient and his family refused. The code status was then changed to do not resuscitate and do not intubate. The patient passed away from complications associated with COVID-19 on the tenth day of admission. Of note, the patient was not vaccinated for COVID-19. 

## Discussion

The clinical course of COVID-19 can range from mild respiratory symptoms to severe complications such as acute respiratory distress syndrome, acute renal failure, septic shock, severe pneumonia, multiorgan failure, and death [4]. As patients with malignancies are immunosuppressed, by either the disease itself or by treatment, they are at a higher risk of infection with COVID-19 and worsened outcomes compared to the general population. Chronic lymphocytic leukemia, a chronic lymphoproliferative disorder of mature B cells, is the most common leukemia in adults in western populations and is characterized by the expansion of CD5 positive lymphocytes [5]. The general relationship between COVID-19 and hematologic malignancies such as CLL is not well understood. However, a multicenter study by Mato et al. concluded that regardless of the phase of CLL or the treatment status, patients diagnosed with COVID-19 with concurrent CLL are at high risk of death [6]. In many cases, leukocytosis has been associated with severe disease and is known to be a poor prognostic factor in patients diagnosed with COVID-19, but hyperleukocytosis has been rarely documented [7]. To our knowledge, there have been less than three reported cases of COVID-19-induced hyperleukocytosis, and therefore little is known about its pathogenesis. 

The reason this case is interesting is that less than two weeks ago the patient was normal with a slightly elevated WBC count and lymphocyte count and did not have lymphadenopathy on examination. However, when he presented with signs and symptoms of COVID-19 he simultaneously had lymphadenopathy on examination and the CT scan with a hyper elevated WBC count and lymphocyte count. The coexistence of COVID-19 and CLL has not been studied well, and a review of four patients with treatment-naive CLL done by Paneesha et al. reported that lymphocyte counts increased by three-fold compared to baseline during their COVID-19 infection [8]. The authors postulate that COVID-19 co-infection with CLL can increase the clonality of B cells. As in a similar case report by Ali et al. a possible scenario is that the patient had monoclonal B-cell lymphocytosis that accelerated into frank CLL by COVID-19 [2]. The other possibility is that indolent CLL became unmasked by COVID-19 producing hyperleukocytosis. 

Pseudohyperkalemia in CLL is found with elevated leukocyte counts due to cell lysis with the consequent release of intracellular potassium but with normal potassium level in vivo. In our case, we believe pseudohyperkalemia developed due to the overwhelming number of leukocytes with increased fragility. The cause of cell destruction may be due to clotting or mechanical trauma with the use of vacuum tubes, prolonged storage, pneumatic tube transport, tourniquet use, repeated fist clenching, and centrifugation [9]. Pseudohyperkalemia in leukocytosis can be seen in both serum and plasma samples unlike in thrombocytosis where it is present mainly in the serum [10]. According to Lee et al., heparinized tubes should not be utilized in cases of high leukocyte counts [10]. In most cases, elevated potassium will be found in serum rather than in plasma. In a study done by Handy et al. at the M.D. Anderson Cancer Center, plasma was found to be the specimen of choice for checking potassium levels [11]. Another clue to the diagnosis of pseudohyperkalemia is the wide variability of potassium measurements on repeat draws. An accurate assessment of potassium can be made by allowing for the separation of serum from cells before centrifugation as the fibrin clot can protect the cells from lysis [12]. Pseudohyperkalemia should be thought of in any asymptomatic patient without apparent cause of hyperkalemia and lack of ECG manifestations with an elevated leukocyte count. 

## Conclusions

This case highlights that COVID-19 can lead to hyperleukocytosis in patients with undiagnosed CLL. In any patient with elevated leukocyte counts above 100 k/cumm who presents with COVID-19, the possibility of concurrent CLL should be looked for. The hyperleukocytosis can in turn lead to pseudohyperkalemia due to an increase in cell fragility in CLL leading to inaccurate potassium values. Physicians should be aware of this phenomenon and we believe that hospitals should flag elevated potassium values in patients with hyperleukocytosis as this may prevent unnecessary treatment with potassium lowering agents. This case highlights our lack of complete understanding of COVID-19 and its multitude of effects. More research has to be done about the effects of COVID-19 on indolent CLL in terms of the cause of hyperleukocytosis, prevention, treatment, and prognosis.
